# The limited storage capacity of gonadal adipose tissue directs the development of metabolic disorders in male C57Bl/6J mice

**DOI:** 10.1007/s00125-015-3594-8

**Published:** 2015-05-12

**Authors:** Lianne van Beek, Jan B. van Klinken, Amanda C. M. Pronk, Andrea D. van Dam, Eline Dirven, Patrick C. N. Rensen, Frits Koning, Ko Willems van Dijk, Vanessa van Harmelen

**Affiliations:** Department of Human Genetics, Leiden University Medical Center, Einthovenweg 20, PO Box 9600, 2300 RC Leiden, the Netherlands; Einthoven Laboratory for Experimental Vascular Medicine, Leiden University Medical Center, Leiden, the Netherlands; Department of Medicine, Division of Endocrinology, Leiden University Medical Center, Leiden, the Netherlands; Department of Immunohematology and Blood Transfusion, Leiden University Medical Center, Leiden, the Netherlands

**Keywords:** Adipocyte size, Crown-like structure, Inflammation, Obesity, Visceral, White adipose tissue

## Abstract

**Aims/hypothesis:**

White adipose tissue (WAT) consists of various depots with different adipocyte functionality and immune cell composition. Knowledge of WAT-depot-specific differences in expandability and immune cell influx during the development of obesity is limited, therefore we aimed to characterise different WAT depots during the development of obesity in mice.

**Methods:**

Gonadal WAT (gWAT), subcutaneous WAT (sWAT) and mesenteric WAT (mWAT) were isolated from male C57Bl/6J mice with different body weights (approximately 25–60 g) and analysed. Linear and non-linear regression models were used to describe the extent of WAT depot expandability and immune cell composition as a function of body weight.

**Results:**

Whereas mouse sWAT and mWAT continued to expand with body weight, gWAT expanded mainly during the initial phase of body weight gain. The expansion diminished after the mice reached a body weight of around 40 g. From this point on, gWAT crown-like structure formation, liver steatosis and insulin resistance occurred. Mouse WAT depots showed major differences in immune cell composition: gWAT consisted mainly of macrophages, whereas sWAT and mWAT primarily contained lymphocytes.

**Conclusions/interpretation:**

Marked inter-depot differences exist in WAT immune cell composition and expandability. The limited storage capacity of gWAT seems to direct the development of metabolic disorders in male C57Bl/6J mice.

**Electronic supplementary material:**

The online version of this article (doi:10.1007/s00125-015-3594-8) contains peer-reviewed but unedited supplementary material, which is available to authorised users.

## Introduction

White adipose tissue (WAT) is the main energy storage organ, and is distributed over various depots. The regional distribution and inflammatory status of WAT are strongly associated with the development of metabolic disorders. Excessive abdominal fat, or central obesity, is known to be a strong risk factor for type 2 diabetes mellitus and cardiovascular disease [1, 2]. WAT can be divided into subcutaneous WAT (sWAT) and visceral WAT (vWAT), located underneath the skin and around the abdominal organs, respectively. Mouse vWAT is generally subdivided into mesenteric WAT (mWAT; between the organs) and gonadal WAT (gWAT; around the testes). While WAT was originally considered an organ with homogeneous function, vWAT is now thought to exert more adverse effects on health compared with sWAT [2–4]. These pathophysiological differences in WAT depots are linked to the metabolic and inflammatory status of the tissue.

Due to excessive fat accumulation in WAT during obesity, adipocytes become stressed and release increased amounts of fatty acids and pro-inflammatory adipokines and chemokines. These inflammatory signals induce immune cell infiltration and dysfunction of the obese WAT [5–7]. Macrophage accumulation, or more specifically the presence of crown-like structures (CLS), in the WAT is associated with adipocyte death caused by cellular lipid overload [8, 9]. Furthermore, T and B lymphocytes are increased in WAT during obesity and contribute to the development of metabolic disorders [10, 11]. Pro-inflammatory cytokines produced by both adipocytes and infiltrating immune cells directly interfere with the insulin signalling pathway, thereby affecting insulin sensitivity both locally and systemically, leading to insulin resistance (IR) and type 2 diabetes [12, 13]. Compared with sWAT, vWAT secretes more fatty acids and pro-inflammatory cytokines and has a higher infiltration of cytotoxic T cells and macrophages during obesity [14–16].

Most of the human studies on WAT inflammation compare WAT between lean and obese individuals and consider only one WAT depot. The majority of mouse studies use male C57Bl/6J mice as a model for obesity induced by a high-fat diet (HFD) and assess only gWAT, whereas sWAT and mWAT are neglected [17]. As different WAT depots have different functions and cellular composition, it is of importance to determine the functional and immunological phenotypes of the various WAT depots. Moreover, longitudinal studies following the development of obesity are sparse and, as a consequence, the inflammatory response of the different WAT depots during body weight gain has, until now, been poorly characterised. Therefore, the aim of the current study was to phenotype the different WAT depots and to determine regional differences with regard to WAT expandability and inflammation in male C57Bl/6J mice during the development of HFD-induced obesity. In addition, we set out to develop a set of linear and non-linear regression models to describe organ weights and WAT (immune cell) composition as a function of body weight.

## Methods

### Animals

Experiments were performed with six different batches of male C57Bl/6J mice (Charles River, Maastricht, the Netherlands). The batches differed in duration (4–34 weeks) and type of HFD (45% or 60% energy derived from lard fat; D12451 or D12492, Research Diet Services, Wijk bij Duurstede, the Netherlands) (electronic supplementary material [ESM] Table 1). Body weight was measured and lean and fat mass was assessed by MRI-based body composition analysis (Echo MRI, Echo Medical Systems, Houston, TX, USA). At the end of the diet intervention, mice were killed, perfused and organs were dissected for further analysis. All experiments were approved by the animal ethics committee of Leiden University Medical Center.

### Adipocyte and stromal vascular cell isolation

Depots of gWAT (one side), sWAT (posterior, one side) and mWAT were dissected from the mice and kept in PBS after the diet intervention. Tissues were processed for adipocyte size determination as previously described [18]. Adipocyte number per fat pad was calculated from the fat pad mass and adipocyte size. The residue of the WAT filtrate was used for the isolation of stromal vascular fraction (SVF) to analyse immune cell composition using flow cytometry. After centrifugation (350 *g*, 10 min) the supernatant fraction was discarded and the pellet was treated with erythrocyte lysis buffer, after which the cells were counted using an automated cell counter (TC10, Bio-Rad, Berkeley, CA, USA). The SVF was fixed using 0.5% (vol./vol.) paraformaldehyde, stored in FACS buffer (PBS, 0.02% (vol./vol.) sodium azide, 0.5% (vol./vol.) FCS) in the dark at 4°C and analysed within 1 week.

### Additional analyses

Plasma, liver triacylglycerol (TG), adipocyte lipolysis, histology and flow cytometry analysis were performed as described in ESM Methods.

### Statistics

Data are presented as single data points or mean ± SD. Statistical differences between groups were calculated with the Student’s *t* test using GraphPad Prism version 6 (GraphPad software, San Diego, CA, USA). Correlation analyses were performed by making correlation plots of body weight vs the variables measured in this study. We modelled the association between each variable and body weight using regression assuming either a linear (*y* = *b* × *x* + *c*; *a* = 1) or non-linear power function. The non-linear power functions could either have *y*-intercept (*y* = *b* × *x*^a^ + *c*; *a* > 1, exponential form), or *x*-intercept (*y* = *b* × (*x* − *c*)^a^; *a* < 1, curve tapering off); *x* = body weight, *y* = lean, fat or individual organ mass. For each analysis the *p* value zero slope (*p*) indicated if the slope was significantly different from 0 (*b* = 0, horizontal line). In addition, superiority of the non-linear (power) function over the linear model was determined by testing the hypothesis *a* = 1 using the extra sum-of-squares *F* test; the corresponding *p* value was termed *p* value linear indicated by *p*^lin^. A Spearman rank correlation coefficient (*r*) was determined for every association. A value of *p* < 0.05 was considered statistically significant, *p* values were corrected by Bonferroni multiple test correction when indicated; significant values in the tables are identified by *p < 0.05, **p < 0.01, ***p < 0.001.

## Results

### HFD-induced changes in body composition and organ weight

**Fig. 1:**
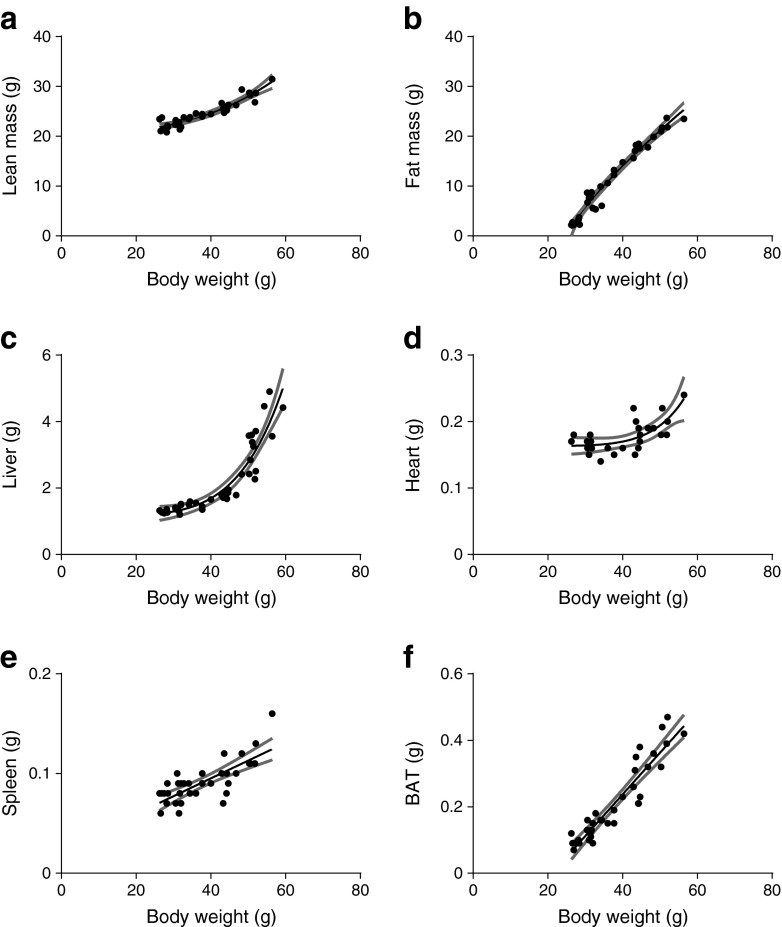
Correlation of HFD-induced changes in body composition and organ weight with body weight. Correlations with body weight in male C57Bl/6J mice (ranging from approximately 25 to 60 g) are shown for lean mass (**a**), fat mass (**b**), and different organ weights: liver (**c**), heart (**d**), spleen (**e**) and interscapular BAT (**f**). Associations were modelled using either a linear model or non-linear function, 95% CIs are shown as grey bands. See ESM Table 2 for equations, correlations and *p* values

In order to study mice with a broad range of body weights, ranging from lean to severely obese (26.3–59.3 g, *n* = 54), C57Bl/6J mice were subjected to different diet interventions for a different number of weeks and with a varying fat percentage in the diet. Lean mice (<30 g) consisted of about 80% lean mass and 20% fat mass. Mice exposed to HFD increased both lean and fat mass, though fat mass increased relatively more (Fig. 1a, b; ESM Table 2). Obese mice consisted of up to 50% fat mass. Figure 1c shows that the liver weight increased non-linearly with a power >1 when correlated with body weight, with a substantially increased liver weight from approximately 40 g upwards. This was mainly caused by an increase of fat in the liver (ESM Fig. 1a). Also, heart weight had a non-linear correlation with body weight (Fig. 1d). Spleen weight correlated linearly with body weight (Fig. 1e), as did brown adipose tissue (BAT), which showed a very strong linear positive correlation with body weight (Fig. 1f). BAT lipid droplet content correlated positively both with body weight (ESM Fig. 1b) and BAT weight (*r* = 0.64, *p* = 0.0001, data not shown).

**Fig. 2:**
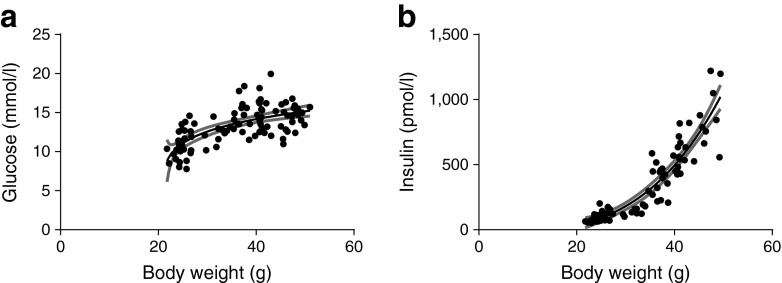
Correlation of HFD-induced changes in plasma glucose and insulin levels with body weight. The correlation between body weight and plasma glucose (**a**, *r* = 0.67, *p* < 0.0001, *p* ^lin^ = 0.0044) and insulin (**b**, *r* = 0.93, *p* < 0.0001, *p* ^lin^ < 0.0001) levels in male C57Bl/6J mice. Associations were modelled using either a linear model or non-linear function; 95% CIs are shown as grey bands. A significant *p* value provides evidence of a non-zero slope in the linear model; a significant *p* ^lin^ value provides evidence that the association is non-linear

ESM Table 2 shows the equations of the curves of the correlations between the individual organs and body weight after best-fit comparison statistics. Plasma glucose and insulin as well as plasma lipid levels were measured and correlated with body weight. Glucose increased at the start of body weight gain, after which it tapered off (Fig. 2a). As glucose levels are regulated by insulin, the flattening of the glucose curve can be linked to increasing insulin levels (Fig. 2b). Plasma total cholesterol correlated positively with body weight (*r* = 0.72, *p* < 0.0001), whereas other plasma lipids (TG and NEFA) did not correlate with body weight (data not shown).

### Expandability of mouse WAT depots

**Fig. 3:**
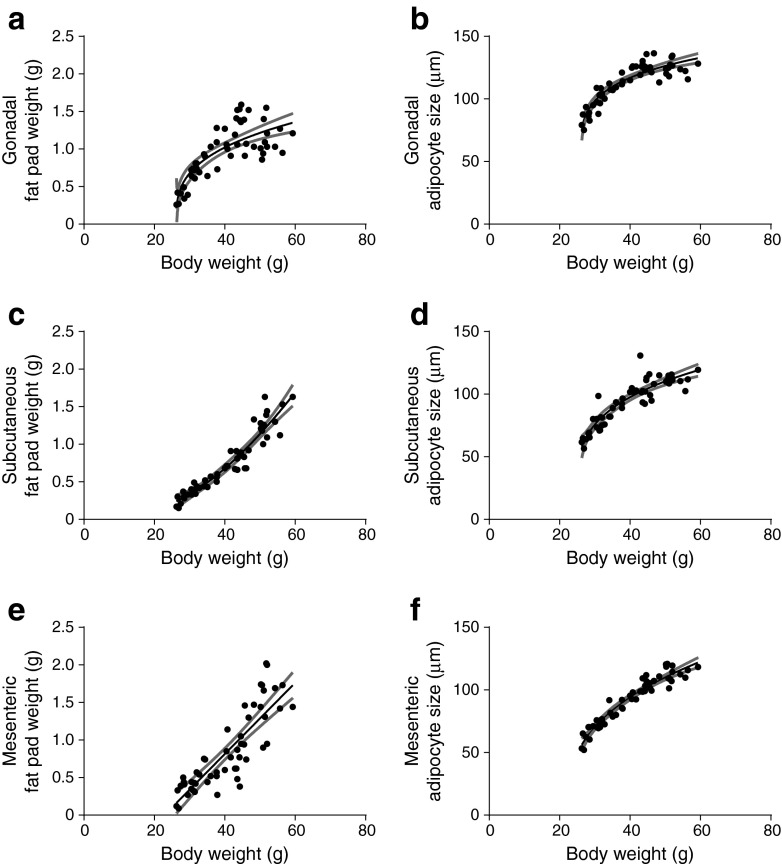
Correlation of HFD-induced changes in fat pad weight and adipocyte size with body weight. Fat pad weight (**a**, **c**, **e**) and adipocyte size (**b**, **d**, **f**) of gWAT, sWAT and mWAT in relation to body weight in male C57Bl/6J mice. For gWAT and sWAT, one fat pad is representative. Associations were modelled using either a linear model or non-linear function; 95% CIs are shown as grey bands. See Table 1 and ESM Table 2 for equations, correlations and *p* values

**Fig. 4:**
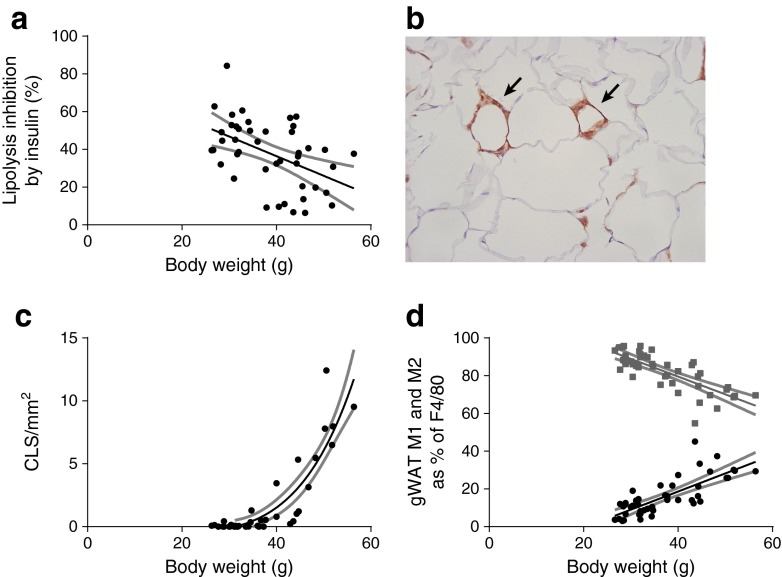
Correlation of insulin responsiveness of adipocytes and macrophage phenotype in gWAT with body weight. Percentage inhibition of lipolysis by insulin of gonadal adipocytes correlated with body weight (**a**, *r* = −0.47, *p* = 0.0010). Insulin responsiveness of the adipocytes was determined by measuring the response of the adipocytes to 8-bromo-cAMP-stimulated lipolysis and the percentage inhibition thereof by insulin. F4/80-stained macrophages in gWAT (**b**), a ×20 magnification is used, CLS are indicated by arrows. CLS counts/mm^2^ WAT correlated with body weight of male C57Bl/6J mice (**c**, *r* = 0.71, *p* < 0.0001, *p* ^lin^ < 0.0001). Macrophage type 1 (CD11B^+^CD11C^+^; black circles; *r* = 0.77, *p* < 0.0001) and 2 (CD11B^+^CD11C^−^; grey squares; *r* = −0.76, *p* < 0.0001) as percentage of F4/80^+^ cells in SVF of gWAT by flow cytometry (**d**) correlated with body weight of male C57Bl/6J mice. Associations were modelled using either a linear or non-linear function; 95% CIs are shown as grey bands. A significant value of *p* provides evidence of a non-zero slope in the linear model; a significant value of *p* ^lin^ provides evidence that the association is non-linear

Fat pad weight of the various WAT depots reflects expandability during HFD exposure. The gWAT depot expanded mostly during the initial phase of weight gain compared with both sWAT and mWAT. With progressing weight gain, the gWAT growth curve tapered off, whereas sWAT and mWAT continued to grow with increasing body weight (Fig. 3a, c, e; ESM Table 2). This is also illustrated by ESM Fig. 2, which shows the results when mice were divided into groups based on body weight to determine the adipocyte size distribution. Whereas for both sWAT and mWAT the adipocyte size distribution curve shifted towards larger adipocytes with higher body weight, gWAT adipocytes remained comparable in size from approximately 40 g onwards. Interestingly, the gonadal adipocytes were larger compared with adipocytes of sWAT and mWAT for both lean and obese mice (Fig. 3b, d, f; ESM Table 3, 4). The potency of insulin to inhibit lipolysis in gonadal adipocytes was tested ex vivo and revealed a negative correlation with body weight (Fig. 4a), as well as with adipocyte size (*r* = −0.42, *p* = 0.0043, data not shown).

WAT growth is accomplished by hypertrophy (increase in size) or hyperplasia (increase in number) of the adipocytes. For all three WAT depots, there was a significant correlation between body weight and adipocyte size (Fig. 3b, d, f; Table 1), whereas adipocyte number did not correlate with body weight (Table 1). However, when correlated with WAT depot weight, adipocyte number did show a slight positive correlation for gWAT and mWAT (data not shown). These data indicate that WAT expansion occurred predominantly by adipocyte hypertrophy and somewhat by hyperplasia in gWAT and mWAT, whereas sWAT expanded only by adipocyte hypertrophy.

**Table 1:** WAT depot composition correlated with body weight of mice on an HFD

Variable	Correlation with body weight			Statistics		
	*a*	*b*	*c*	*r*	*p* value^a^ Zero slope	*p* value^a^ Linear
gWAT						
Adipocyte size (μm; *n* = 54)	0.1482	78.63	25.46	0.8112	1.40 × 10^−19^***	2.92 × 10^−07^***
Adipocyte no./fat pad (*n* = 46)				0.1822	1.000	–
SVF no.^b^/fat pad (*n* = 54)	1	40,829	−672,214	0.6768	1.20 × 10^−05^***	1.000
Leucocytes (% CD45 of SVF; *n* = 44)	1	0.5060	42.98	0.4131	0.1197	–
T lymphocytes (% CD3 of SVF; *n* = 44)	1	0.2878	0.1631	0.3518	0.0315*	1.000
T lymphocyte ratio (CD4:CD8; *n* = 51)	1	−0.1689	10.71	−0.6287	7.28 × 10^−05^***	0.8226
B lymphocytes (% CD19 of SVF; *n* = 35)	1	0.1398	−1.206	0.3631	0.0963	–
Macrophages (% F4/80 of SVF; *n* = 50)				−0.1962	1.000	–
Macrophage ratio (M1:M2; *n* = 45)	1	0.01534	−0.3574	0.7710	7.92 × 10^−08^***	1.000
sWAT						
Adipocyte size (μm; *n* = 54)	0.2473	49.44	24.39	0.8886	3.26 × 10^−20^***	4.33 × 10^−04^***
Adipocyte no./fat pad (*n* = 46)				−0.03627	1.000	–
SVF no.^a^/fat pad (*n* = 54)				0.2907	1.000	–
Leucocytes (% CD45 of SVF; *n* = 50)	1	0.6146	32.41	0.4680	0.0036**	1.000
T lymphocytes (% CD3 of SVF; *n* = 40)				0.1068	1.000	–
T lymphocyte ratio (CD4:CD8; *n* = 50)	1	−0.03013	2.784	−0.4064	0.1818	–
B lymphocytes (% CD19 of SVF; *n* = 43)				−0.07501	1.000	–
Macrophages (% F4/80 of SVF; *n* = 29)				−0.1822	1.000	–
Macrophage ratio (M1:M2; *n* = 34)	1	0.01025	−0.2255	0.6262	1.95 × 10^−06^***	0.5508
mWAT						
Adipocyte size (μm; *n* = 54)	0.3524	34.09	22.17	0.9528	3.17 × 10^−29^***	3.23 × 10^−04^***
Adipocyte no./fat pad (*n* = 46)				−0.06658	1.000	–
SVF no.^a^/fat pad (*n* = 54)				0.1914	1.000	–
Leucocytes (% CD45 of SVF; *n* = 49)	1	−0.6190	97.37	−0.3613	0.0729	–
T lymphocytes (% CD3 of SVF; *n* = 48)	1	−0.7400	54.93	−0.6355	7.07 × 10^−06^***	0.5445
T lymphocyte ratio (CD4:CD8; *n* = 48)				0.06193	1.000	–
B lymphocytes (% CD19 of SVF; *n* = 30)	1	−0.9100	72.53	−0.3696	0.0261	–
Macrophages (% F4/80 of SVF; *n* = 23)	1	0.9423	−22.89	0.5198	0.0153*	0.1161
Macrophage ratio (M1:M2; *n* = 29)	1	0.01035	−0.2701	0.6768	6.93 × 10^−04^***	0.2763

^a^ *p* value after Bonferroni multiple test correction; *p<0.05, **p<0.01, ***p<0.001

^b^Absolute number of cells in the SVF

No. number

### HFD-induced changes in immune cell composition in mouse WAT depots

WAT depots were processed to isolate the SVF, which contains immune cells as well as pre-adipocytes and endothelial cells. The absolute SVF cell number was determined and represented per fat pad. The SVF cell count of gWAT correlated positively with body weight, whereas the SVF cell counts of sWAT and mWAT did not correlate with body weight (Table 1). Absolute leucocyte numbers (CD45^+^ cells) per fat pad showed a linear correlation with body weight for both gWAT and sWAT, whereas leucocyte numbers in mWAT showed no correlation with body weight (ESM Figs 3a, 4a, 5a). Absolute T cell numbers in gWAT and sWAT, but not mWAT, correlated positively with body weight (ESM Figs 3b, 4b, 5b). Within the T cell population, the ratio between T helper cells and cytotoxic T cells (CD4^+^ and CD8^+^ cells, respectively) was determined. In both gWAT and sWAT the CD4:CD8 ratio showed a negative correlation with bodyweight, which indicates a larger relative increase in cytotoxic T cells compared with T helper cells (Table 1). Absolute B cell numbers (CD19^+^ cells) showed a positive correlation with body weight for gWAT (ESM Fig. 3d).

Absolute macrophage numbers (F4/80^+^ cells) of all three WAT depots correlated positively with body weight (ESM Figs 3c, 4c, 5c). Interestingly, absolute macrophage numbers in gWAT and sWAT showed a non-linear correlation with body weight with a power >1, while the correlation in mWAT was linear. WAT macrophages can form CLS, which is shown in Fig. 4b by F4/80 staining of gWAT. CLS increased non-linearly with increasing body weight with a power >1 in the gWAT depot (Fig. 4c). Within the F4/80^+^ cell population, M1 and M2 macrophages were distinguished using CD11B and CD11C markers (M1, CD11B^+^CD11C^+^; M2, CD11B^+^CD11C^−^). Figure 4d shows the correlation of M1 and M2 macrophages as percentages of F4/80 cells from the gWAT depot (representative for the other two depots, data not shown) with body weight. Within all adipose tissue depots, M1 macrophages were positively correlated and M2 macrophages were negatively correlated with body weight. The M1:M2 ratio also showed a strong positive correlation with body weight within all WAT depots (Table 1). This indicates relatively more M1 macrophages in WAT with a higher body weight. Thus, HFD induces immune cell compositional changes in all three WAT depots, with an increase in immune cell numbers mainly in gWAT and sWAT.

### Immune cell composition of distinct WAT depots from lean and obese mice

The gWAT, sWAT and mWAT depots from either lean or obese mice (mean body weight 31.0 ± 2.9 g, *n* = 10 and 50.1 ± 3.6 g, *n* = 8, respectively) were analysed and compared with each other to determine differences in immune cell composition between the adipose tissue regions. In lean mice, approximately 60% of the SVF from gWAT and sWAT consisted of leucocytes (57.5 ± 9.9% and 62.7 ± 6.2%, respectively), and in mWAT this percentage was even higher (78.3 ± 12.1%) (ESM Table 3). In obese mice, there were no differences in leucocyte percentage in the SVF between the different WAT depots (approximately 65%, ESM Table 4). T cells were present in all three WAT depots. Interestingly, in mWAT from lean mice the percentage of T cells in the SVF was significantly higher compared with gWAT and sWAT (ESM Table 3). The CD4:CD8 ratio in lean sWAT and mWAT was between 1 and 2, which indicates slightly more CD4 cells than CD8 cells. However, lean gWAT contained even more CD4 than CD8 cells as the ratio was around 5 (ESM Table 3). In obese mice, there were no differences in T cell percentages between the WAT depots (ESM Table 4). There were major differences in B cell content between the depots, ranging from hardly any B cells in gWAT to approximately 35% of the SVF in mWAT of lean mice and approximately 20% of the SVF in mWAT of obese mice (ESM Tables 3 and 4). The gWAT predominantly contained macrophages (approximately 30% of SVF both in lean and obese mice), while less than 10% of the SVF from sWAT consisted of macrophages in both lean and obese mice. These data indicate that there are large differences in immune cell composition between different WAT depots from both lean and obese mice.

## Discussion

In the current study we determined intra-depot differences in WAT immune cell composition in relation to WAT expandability. Mouse WAT depots showed major differences in expandability and immune cell infiltration during the development of obesity. Furthermore, a body weight of approximately 40 g emerged as a critical tipping point from whereon metabolic dysfunction occurred, at least in male C57Bl/6J mice.

It has been extensively shown that distinct WAT depots from both mice and humans have different metabolic functions. This is due to intrinsic differences in adipocyte characteristics but has also been attributed to differences in immune cell composition in the various depots [19–21]. Here, we have performed a direct comparison of the different WAT depots in male C57Bl/6J mice and focussed simultaneously on the expandability and immune cell composition during the development of obesity, which has until now been poorly characterised. Our data confirm great variability in immune cell composition between WAT depots. The characteristics of the different mouse WAT depots already differed in the lean state, and each depot responded differently to body weight gain with respect to immune cell composition as well as expandability. Mouse gWAT expanded mostly during the initial phase of body weight gain, and increased less after a body weight of around 40 g. Although sWAT and mWAT did not primarily expand as fast as gWAT, they both kept expanding with body weight after 40 g. This implies that gWAT is the primary storage depot that grows initially in HFD-induced obesity, followed by sWAT and mWAT. This is also reflected by the larger gWAT adipocytes seen during both the lean and obese states, and which have also been identified by Sackmann-Sala et al, albeit only in lean mice [22].

At around a body weight of 40 g, at which point the gWAT growth curve tapered off, the liver started to grow significantly, mainly caused by an increase in fat content. Apparently, the gWAT adipocytes were saturated and could not grow any larger to store additional fat. As a consequence, the excess fat that could not be stored in the gWAT depot was stored ectopically in the liver [23]. Moreover, around this point of body weight gain, the number of CLS started to increase in the gWAT. As CLS are found around stressed and dying adipocytes [9], the increase in numbers of CLS around 40 g body weight appear to be associated with increased adipocyte death. Also, insulin levels increased substantially from this point on, indicating the development of insulin resistance. Our data therefore imply that approximately 40 g body weight is an important tipping point in male C57Bl/6J mice from whereon WAT and systemic metabolic dysfunction occur concomitantly. In this study, data have been exclusively obtained from male C57Bl/6J mice in combination with HFD to induce obesity. Whether females, other mouse strains/models or humans also have such a threshold BMI at which WAT inflammation and metabolic dysfunction rapidly increase remains to be investigated.

Our data are in agreement with the study of Strissel et al, which also showed that at a certain body weight in mice gWAT stops expanding because of increased adipocyte death, whereas liver starts accumulating fat [24]. Strikingly, while our data showed a constant gWAT weight and continuous increase in CLS, they showed a reduction in both gWAT weight and CLS formation after reaching a body weight of 40 g. This was accompanied by a reduction in adipocyte size and increased adipocyte numbers, which they attributed to newly differentiated adipocytes. One explanation for their findings might be that they fed their mice a 60% HFD for 20 weeks, which may be a more extreme intervention than those used in our study. There are several other studies elucidating regional differences in WAT growth [25–27], although these studies do not extensively link WAT expandability to immune cell infiltration and metabolic characterisation. This study is the first that focusses on all these processes simultaneously.

The size of adipocytes increased in all three WAT depots during body weight gain. Adipocyte number per fat pad remained comparable in all depots when correlated with body weight, whereas in gWAT and mWAT adipocyte numbers increased slightly during the HFD intervention when correlated with fat pad weight. Our observations are in line with previous mouse studies that showed expansion by hypertrophy only in sWAT, whereas mWAT expanded by both hypertrophy and hyperplasia [25, 27]. Adipocytes in gWAT were larger than those in sWAT and mWAT in mice. Large adipocytes are thought to release more pro-inflammatory cytokines and chemokines that attract circulating monocytes into the WAT which, in turn, differentiate into macrophages [5, 28]. Indeed, the SVF of the gWAT contained a higher fraction of macrophages compared with sWAT and mWAT. Also, the absolute number of macrophages per WAT depot was higher in the gWAT than in the other WAT depots.

The absolute leucocyte numbers in mouse mWAT were much higher compared with sWAT and gWAT. The mWAT surrounds the intestine, which represents the first line of defence against intestinal pathogens and could therefore explain the large number of leucocytes. However, mWAT is also known to contain a large amount of lymphoid tissue including lymph nodes and milky spots [29]. Although we took great care to remove all visible lymph nodes from the mWAT before the immune cell characterisation, we cannot exclude the possibility that we missed some lymph nodes. As lymph nodes contain numerous leucocytes, this could also explain the high number of leucocytes present in mouse mWAT. Another issue affecting analyses of WAT inflammation might be contamination of the SVF with immune cells from the circulation. However, our mice were perfused before removal of the WAT depots.

Numerous pathophysiological processes are known to be associated with the development of IR. In this study, we focussed on WAT expendability and inflammation as a measure for WAT dysfunction. However, inadequate angiogenesis and related hypoxia are known as early determinants for WAT dysfunction as well [30, 31], and can induce WAT fibrosis, which has also been associated with IR [32]. Although beyond the scope of the current study, it is interesting to determine the association between these pathologies, WAT expansion and inflammation. Macrophages are highly abundant in WAT with, specifically, M1 macrophages accumulating during obesity and contributing to IR. Our data showed a phenotypic switch from M2 to M1 type macrophages during obesity, which has previously also been shown by Lumeng et al [17]. The number of CLS, which are primarily formed by M1 macrophages, also increased with body weight [33]. M1 macrophages are known to accumulate lipids and form foam cells. Lipid accumulation in macrophages has previously been directly related to the expansion of WAT [34]. Whether lipid-loaded macrophages are a consequence of the limited expansion of WAT remains to be investigated.

BAT is known to be a prominent player in body weight control, as it burns TG to produce heat [35, 36]. Here, we show that BAT weight is strongly correlated with body weight. In general, high BAT weight is associated with inactive BAT, as TG is being stored instead of being used for heat production [37]. This is supported by an increased lipid droplet content in BAT with higher body weight. Our observed correlation between BAT weight and body weight can be explained by the thermal insulation function of WAT. The increased WAT in obesity is enough to keep the animal warm and heat production by BAT activity is reduced. Thus, body weight is an important confounder when studying BAT activity.

We conclude that mouse WAT depots vary considerably in expandability and immune cell composition during HFD-induced body weight gain. With a body weight threshold of approximately 40 g in mice, gWAT seems to have reached its maximum expansion capacity and at this point WAT dysfunction and concomitant systemic metabolic dysfunction will commence.

## Electronic supplementary material

ESM Methods (PDF 300 kb)

ESM Fig. 1 (PDF 376 kb)

ESM Fig. 2 (PDF 353 kb)

ESM Fig. 3 (PDF 604 kb)

ESM Fig. 4 (PDF 577 kb)

ESM Fig. 5 (PDF 546 kb)

ESM Table 1 (PDF 192 kb)

ESM Table 2 (PDF 424 kb)

ESM Table 3 (PDF 421 kb)

ESM Table 4 (PDF 422 kb)

## Abbreviations

BAT: Brown adipose tissue
CLS: Crown-like structures
gWAT: Gonadal white adipose tissue
HFD: High-fat diet
IR: Insulin resistance
mWAT: Mesenteric white adipose tissue
SVF: Stromal vascular fraction
sWAT: Subcutaneous white adipose tissue
TG: Triacylglycerol
WAT: White adipose tissue
