# Supplementary material for: The limited storage capacity of gonadal adipose tissue directs the development of metabolic disorders in male C57Bl/6J mice
Source: Diabetologia. 2015 May 12;58(7):1601–9. doi: 10.1007/s00125-015-3594-8 (PMC4473015; doi:10.1007/s00125-015-3594-8)
Supplement: Supplementary file 1 — (PDF 300 kb) [file 125_2015_3594_MOESM1_ESM.pdf]

ESM methods

Plasma parameters

At different time points during the HFD intervention blood was drawn from 6 hour fasted mice via the tail vein into paraoxon (Sigma, St. Louis, MO) coated capillary tubes to prevent ongoing *in vitro* lipolysis. After centrifugation, plasma was collected and glucose, insulin, triacylglycerol (TG), total cholesterol, and non-esterified fatty acid were determined using commercially available kits (Instruchemie, Delfzijl, The Netherlands; Crystal Chem Inc., IL, USA; 11488872 and 236691, Roche Molecular Biochemicals, Indianapolis; NEFA-C, Wako chemicals GmbH, Neuss, Germany, respectively).

Liver TG analysis

Lipids were extracted from livers according to a modified protocol from Bligh and Dyer [1]. Briefly, small liver pieces were homogenized in ice-cold methanol. Lipids were extracted by addition of 1800 µl ice-cold chloroform/methanol (3:1) to 45 µl homogenate. The chloroform/methanol phase was dried and dissolved in 2% (vol./vol.) Triton X-100. Hepatic TG concentrations were measured using TG kit (11488872, Roche Molecular Biochemicals, Indianapolis). Liver TG were expressed per mg protein, which was determined using the BCA protein assay kit (Pierce Biotechnologie, Rockford, USA).

Adipocyte lipolysis assay

The potency of insulin to inhibit lipolysis in gonadal adipocytes was determined by incubating isolated adipocytes (±10,000 cells/ml) for 2 h at 37°C, with DMEM/F12 with 2% (vol./vol.) BSA and 8-bromo-cAMP (10^{-3} mol/l; Sigma, St. Louis, MO) in combination with or without insulin (10^{-9} mol/l). Glycerol concentrations were determined as a measure for lipolysis, using a free glycerol kit (Sigma, St. Louis, MO) and the hydrogen peroxide sensitive fluorescence dye Amplex Ultra Red, as previously described by Clark et al. [2].

Histology

Formalin fixed and paraffin embedded sections of gWAT and intrascapular BAT were used for histological analysis. An F4/80+ antibody (1:250) (Leiden University Medical Center, Leiden, The Netherlands) was used to stain macrophages in gWAT. Vectastain ABC (Vector laboratories, CA, US) was used for visualization of the antibody complex according to manufacturer's instructions. Haematoxylin staining of the gWAT and BAT sections was done using a standard protocol. The area of intracellular lipid vacuoles in BAT was quantified using Image J (NIH, US).

Flow cytometry analysis

Mouse SVF cells were stained with fluorescently labelled antibodies for CD45.2-FITC (BioLegend), CD3-APC, CD4-Qdot605, CD8a-PercpCy5.5, CD19-PE, F4/80-PE, CD11B-PB, CD11C-APCCy7 (all purchased from eBioscience, CA, USA or BioLegend, CA, USA). Cells were measured on a LSR II flow cytometer (BD Biosciences). Data were analysed using FlowJo software (Treestar, OR, USA).

Reference list

[1] Bligh EG, Dyer WJ (1959) A rapid method of total lipid extraction and purification. Canadian journal of biochemistry and physiology 37: 911-917

[2] Clark AM, Sousa KM, Jennings C, MacDougald OA, Kennedy RT (2009) Continuous-flow enzyme assay on a microfluidic chip for monitoring glycerol secretion from cultured adipocytes. Analytical chemistry 81: 2350-2356
