# Supplementary material for: The limited storage capacity of gonadal adipose tissue directs the development of metabolic disorders in male C57Bl/6J mice
Source: Diabetologia. 2015 May 12;58(7):1601–9. doi: 10.1007/s00125-015-3594-8 (PMC4473015; doi:10.1007/s00125-015-3594-8)

ESM Figure 1. Liver TG and BAT lipid droplet content. Liver TG content (A) and BAT lipid droplet content (B) are depicted per body weight. Associations were modelled using either a linear or non-linear function, 95% confidence interval is shown as grey bands. A significant value of *p* provides evidence of a non-zero slope in the linear model; a significant value of *p** provides evidence that the association is non-linear.

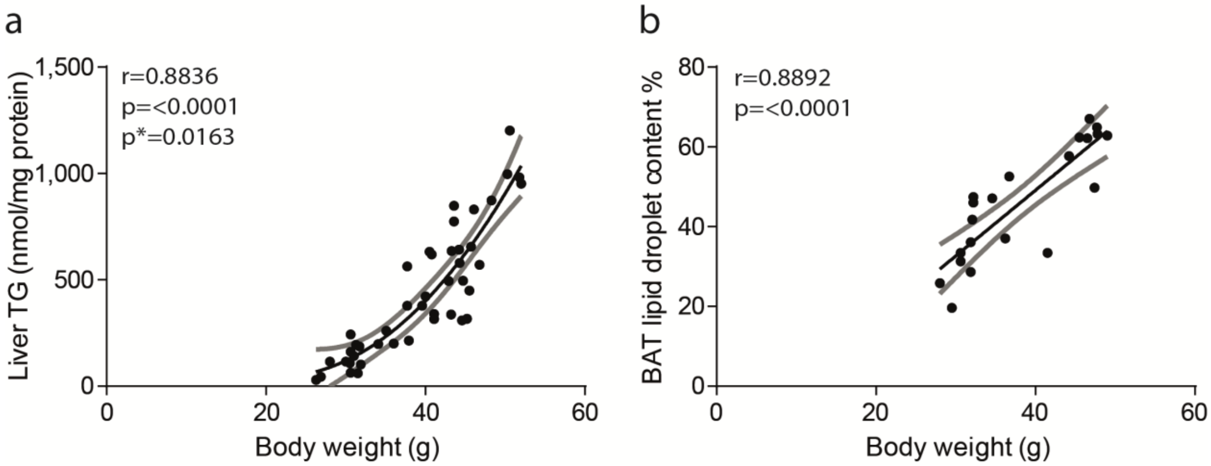

Supplement: Supplementary file 2 — (PDF 376 kb) [file 125_2015_3594_MOESM2_ESM.pdf]
