# Supplementary material for: The limited storage capacity of gonadal adipose tissue directs the development of metabolic disorders in male C57Bl/6J mice
Source: Diabetologia. 2015 May 12;58(7):1601–9. doi: 10.1007/s00125-015-3594-8 (PMC4473015; doi:10.1007/s00125-015-3594-8)

**ESM Figure 2. Adipocyte size distributions with different body weights depicted per WAT depot.** Mice were subdivided into different body weight groups; grey <30 grams, black 30-40 grams, pink 40-50 grams, red >50 grams. The mean adipocyte size distribution per group was depicted for the different WAT depots; gWAT (A), sWAT (B) and mWAT (C).

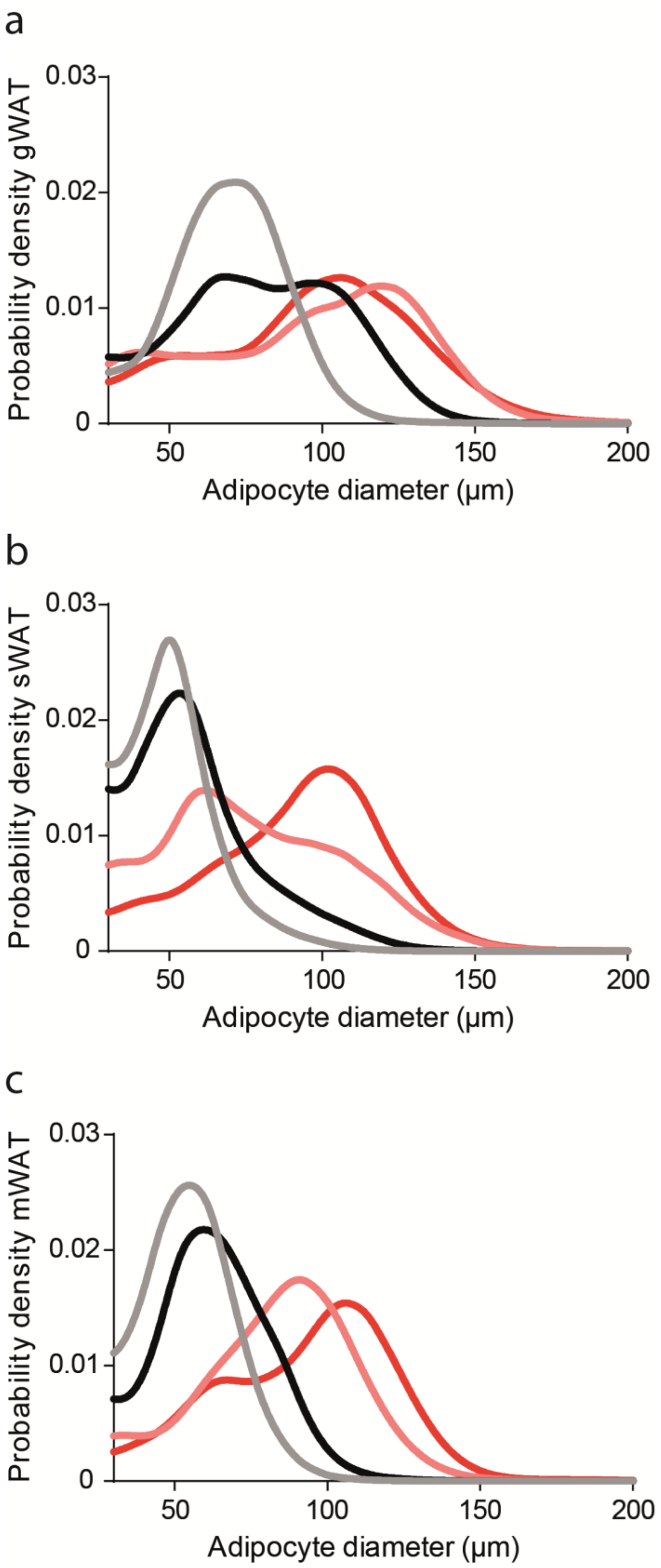

Supplement: Supplementary file 3 — (PDF 353 kb) [file 125_2015_3594_MOESM3_ESM.pdf]
