# Supplementary material for: The limited storage capacity of gonadal adipose tissue directs the development of metabolic disorders in male C57Bl/6J mice
Source: Diabetologia. 2015 May 12;58(7):1601–9. doi: 10.1007/s00125-015-3594-8 (PMC4473015; doi:10.1007/s00125-015-3594-8)

ESM Figure 5. Absolute immune cell numbers per fat pad of mesenteric WAT correlated to body weight. Absolute numbers of leukocytes (A), T lymphocytes (B), macrophages (C), B lymphocytes (D), T helper (black dots) and cytotoxic T lymphocytes (grey squares) (E), and M1 (black dots) and M2 (grey squares) types of macrophages (F) are depicted per body weight. Associations were modelled using either a linear or non-linear function, 95% confidence interval is shown as grey bands. A significant value of *p* provides evidence of a non-zero slope in the linear model; a significant value of *p** provides evidence that the association is non-linear.

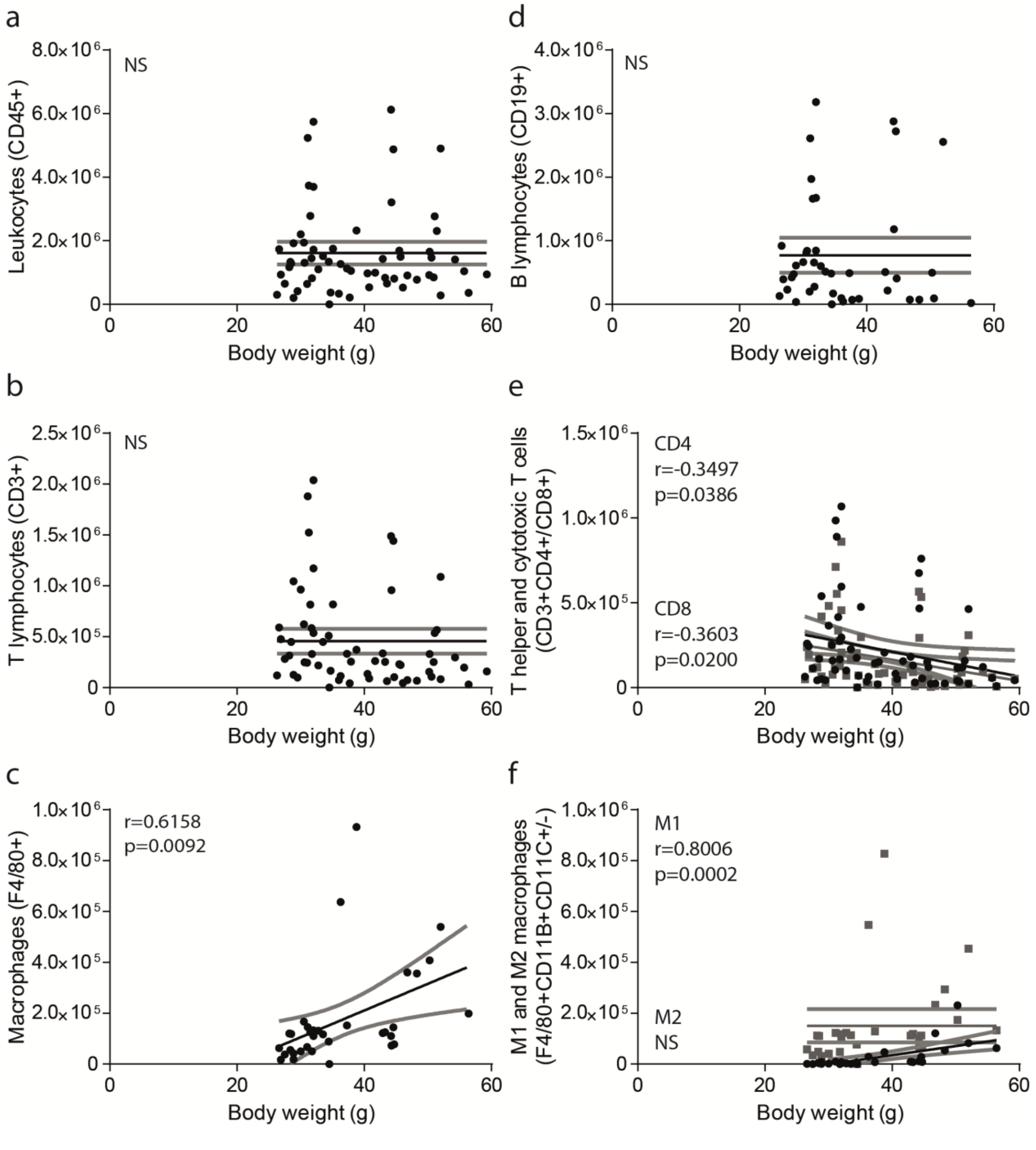

Supplement: Supplementary file 6 — (PDF 546 kb) [file 125_2015_3594_MOESM6_ESM.pdf]
