# Supplementary material for: The limited storage capacity of gonadal adipose tissue directs the development of metabolic disorders in male C57Bl/6J mice
Source: Diabetologia. 2015 May 12;58(7):1601–9. doi: 10.1007/s00125-015-3594-8 (PMC4473015; doi:10.1007/s00125-015-3594-8)
Supplement: Supplementary file 7 — (PDF 192 kb) [file 125_2015_3594_MOESM7_ESM.pdf]

ESM Table 1. Batches of mice with different type and duration of diet intervention

Batch number	Weeks on diet	Type of diet	Number of animals (n)
1	4	45% HFD	10
2	8	45% HFD	10
3	11	45% HFD	9
4	34	45% HFD	8
5	5	60% HFD	9
6	15	60% HFD	8
