# Supplementary material for: The limited storage capacity of gonadal adipose tissue directs the development of metabolic disorders in male C57Bl/6J mice
Source: Diabetologia. 2015 May 12;58(7):1601–9. doi: 10.1007/s00125-015-3594-8 (PMC4473015; doi:10.1007/s00125-015-3594-8)
Supplement: Supplementary file 8 — (PDF 424 kb) [file 125_2015_3594_MOESM8_ESM.pdf]

ESM Table 2. Body composition and organ weight correlated to body weight of mice on a HFD

	Correlation to body weight - Statistics					
	a	b	c	*r*	*P*-value[a] zero slope	*P*-value[a] linear
**Lean mass (g; n=36)**	2.757	1.536e-004	20.60	0.9059	2.95E-15***	0.1206
**Fat mass (g; n=36)**	0.781	1.738	25.48	0.9517	7.75E-24***	0.1782
**Liver weight (g; n=43)**	5.123	3.160e-009	1.180	0.9424	3.94E-17***	3.12E-06***
**Heart weight (g; n=26)**	6.893	6.024e-014	0.1633	0.5734	2.70E-03**	0.1728
**Spleen weight (g; n=35)**	1	1.786e-003	0.0237	0.7271	5.07E-07***	1.000
**BAT weight (g; 36)**	1	0.01254	0.2645	0.9289	6.24E-16***	1.000
**Gonadal FP weight[b] (g; n=54)**	0.320	0.4411	26.21	0.7254	3.18E-11***	7.73E-04***
**Subcutaneous FP weight[b] (g; n=54)**	1	0.04113	-0.9205	0.9607	4.20E-26***	0.1620
**Mesenteric FP weight (g; n=54)**	1	0.0474	-1.084	0.8765	2.36E-20***	0.7785

Linear correlation: a=1; equation: $y=b \times x+c$

Non-linear power function with y-intercept: a>1; equation: $y=b \times x^a+c$

Non-linear power function with x-intercept: a<1 ; equation: $y=b \times (x-c)^a$

x=body weight; y=lean, fat or individual organ mass.

[a]*p*-value after bonferroni multiple test correction

[b]for gonadal and subcutaneous fat pad (FP) weight one of the two pads was used for calculations
