# Supplementary material for: The limited storage capacity of gonadal adipose tissue directs the development of metabolic disorders in male C57Bl/6J mice
Source: Diabetologia. 2015 May 12;58(7):1601–9. doi: 10.1007/s00125-015-3594-8 (PMC4473015; doi:10.1007/s00125-015-3594-8)
Supplement: Supplementary file 9 — (PDF 421 kb) [file 125_2015_3594_MOESM9_ESM.pdf]

ESM Table 3. Composition and comparison of WAT depots from lean mice

	Mouse WAT			Statistics[a]		
	gWAT (n=10)	sWAT (n=10)	mWAT (n=10)	T-test gWAT *vs* sWAT	T-test gWAT *vs* mWAT	T-test sWAT *vs* mWAT
**Adipocyte size (µm)**	97.8±13.2	77.0±13.1	71.3±11.8	0.0184*	0.0016**	1.000
**Adipocyte no/FP (*10^6)**	2.03±0.70	2.81±0.98	2.69±0.59	0.4440	0.2912	1.000
**SVF nr/FP (*10^6)**	0.50±0.16	0.23±0.11	1.96±1.29	0.0024**	0.0192*	0.0040**
**Leukocytes (% CD45 of SVF)**	62.7±6.2	57.5±9.9	78.3±12.1	1.000	0.0184*	0.0088**
**T lymphocytes (% CD3 of SVF)**	7.5±2.2	14.2±8.9	27.5±10.2	0.2768	9.44E-05***	0.0744
**T lymphocyte ratio (CD4:CD8)**	5.1±2.3	1.54±0.66	1.29±0.36	0.0024**	8.00E-04***	1.000
**B lymphocytes (% CD19 of SVF)**	1.6±0.6	13.5±8.7	36.5±13.3	0.0032**	1.70E-06***	0.0040**
**Macrophages (% F4/80 of SVF)**	29.8±3.0	6.4±2.2	4.7±2.6	5.14E-06***	2.62E-05***	1.000

[a]*p*-value after bonferroni multiple test correction
