# Supplementary material for: The limited storage capacity of gonadal adipose tissue directs the development of metabolic disorders in male C57Bl/6J mice
Source: Diabetologia. 2015 May 12;58(7):1601–9. doi: 10.1007/s00125-015-3594-8 (PMC4473015; doi:10.1007/s00125-015-3594-8)
Supplement: Supplementary file 10 — (PDF 422 kb) [file 125_2015_3594_MOESM10_ESM.pdf]

ESM Table 4. Composition and comparison of WAT depots from obese mice

	Mouse WAT			Statistics[a]		
	gWAT (n=8)	sWAT (n=8)	mWAT (n=8)	T-test gWAT *vs* sWAT	T-test gWAT *vs* mWAT	T-test sWAT *vs* mWAT
**Adipocyte size (µm)**	124.4±8.0	112.0±2.5	113.9±5.5	0.0072**	0.0696	1.000
**Adipocyte no/FP (*10^6)**	2.10±0.36	3.02±0.47	3.41±0.84	0.0048**	0.0096**	1.000
**SVF nr/FP (*10^6)**	1.67±0.45	0.75±0.26	2.79±1.78	0.0016**	0.8328	0.0504
**Leukocytes (% CD45 of SVF)**	63.1±7.9	67.0±3.5	67.9±16.4	1.000	1.000	1.000
**T lymphocytes (% CD3 of SVF)**	6.6±2.0	12.5±4.2	11.3±9.4	0.0368*	1.000	1.000
**T lymphocyte ratio (CD4:CD8)**	1.15±0.47	1.00±0.24	1.77±0.56	1.000	0.3376	0.0280*
**B lymphocytes (% CD19 of SVF)**	2.1±1.2	13.0±10.2	19.3±21.2	0.1224	0.4192	1.000
**Macrophages (% F4/80 of SVF)**	37.5±3.9	6.5±1.8	17.9±10.5	7.69E-11***	0.0032**	0.0792

[a]*p*-value after bonferroni multiple test correction
